# Antiplatelet Agents and Oral Anticoagulant Use in Patients with Atrial Fibrillation and Carotid Artery Disease After First-Time Ischaemic Stroke

**DOI:** 10.1007/s10557-023-07433-4

**Published:** 2023-01-24

**Authors:** Stephanie L. Harrison, Benjamin J.R. Buckley, Deirdre A. Lane, Elnara Fazio-Eynullayeva, Paula Underhill, Andrew Hill, David J. Werring, Gregory Y.H. Lip

**Affiliations:** 1 Liverpool Centre for Cardiovascular Science, University of Liverpool, Liverpool John Moores University and Liverpool Heart & Chest Hospital, William Henry Duncan Building, L7 8TX Liverpool, UK; 2 Department of Cardiovascular and Metabolic Medicine, Institute of Life Course and Medical Sciences, University of Liverpool, Liverpool, UK; 3 Aalborg Thrombosis Research Unit, Department of Clinical Medicine, Aalborg University, Aalborg, Denmark; 4 TriNetX Inc., London, UK; 5 Department of Medicine for Older People, Whiston Hospital, St Helens & Knowsley Teaching Hospitals NHS Trust, Prescot, UK; 6 Stroke Research Centre, UCL Queen Square Institute of Neurology, London, UK

**Keywords:** Stroke, Atrial fibrillation, Carotid artery disease, Antiplatelet agents, Anticoagulants

## Abstract

**Introduction:**

People with atrial fibrillation (AF) frequently have competing mechanisms for ischaemic stroke, including extracranial carotid atherosclerosis. The objective of this study was to determine associations between use of oral anticoagulants (OACs) plus antiplatelet agents (APA) after ischaemic stroke and outcomes for patients with AF and carotid artery disease.

**Patients and Methods:**

A retrospective cohort study was conducted. Participants receiving OACs with or without APA were propensity score–matched for age, sex, ethnicity, co-morbidities and presence of cardiac and vascular implants and grafts. Outcomes were 1-year mortality, recurrent stroke and major bleeding.

**Results:**

Of 5708 patients, 24.1% (*n*=1628) received non-vitamin K antagonist OACs (NOACs) with no APA, 26.0% (*n*=1401) received NOACs plus APA, 20.7% (*n*=1243) received warfarin without APA and 29.2% (*n*=1436) received warfarin plus APA. There was no significant difference in risk of recurrent stroke between the groups. Compared to receiving NOACs without APA, receiving warfarin plus APA was associated with a higher risk of mortality (hazard ratio (HR) 1.51 (95% confidence interval (CI) 1.20, 1.89)) and major bleeding (HR 1.66 (95% CI 1.40, 1.96)). Receiving NOACs plus APA was also associated with a higher risk of major bleeding compared to NOACs without APA (HR 1.27 (95% CI 1.07, 1.51), respectively).

**Conclusions:**

The results suggest for patients with AF and carotid artery disease after ischaemic stroke, receiving NOACs without APA is associated with a lower risk of major bleeding with no negative impact on recurrent stroke or mortality. Evidence from randomised trials is needed to confirm this finding.

**Supplementary Information:**

The online version contains supplementary material available at 10.1007/s10557-023-07433-4.

## Introduction

Atrial fibrillation (AF) is associated with a five-fold higher risk of ischaemic stroke, and a higher risk of stroke recurrence and mortality [1]. The recommended management of AF after ischaemic stroke is to offer oral anticoagulants (OACs) to reduce the risk of recurrent stroke [2].

Observational evidence has suggested carotid artery disease may be associated with a two-fold increased risk of stroke recurrence in patients with AF [3]. AF and carotid disease are both potential causes of ischaemic stroke, and optimal secondary stroke prevention should target reduction in stroke recurrence risk associated with both processes. OACs have not been shown to be effective for reducing athero-thromboembolism in carotid disease, whereas antiplatelet agents (APA) have [4]. Therefore, the addition of APA to OACs to reduce the risk of recurrent stroke for patients with AF and carotid artery disease may be considered in clinical practice; however, there are limited data available on how this approach impacts patient outcomes [2, 5]. This results in a critical management challenge for healthcare professionals treating patients with AF and concomitant carotid artery disease after ischaemic stroke. The long-term use of APA and OACs for patients with AF increases the risk of adverse outcomes including major bleeding and intracerebral haemorrhage (ICH) [6]. Nonetheless, large-vessel athero-thromboembolism, such as that associated with carotid artery stenosis or occlusion, accounts for an estimated 40% of acute ischaemic strokes [7].

Non-vitamin K antagonist (VKA) oral anticoagulants (NOACs; also referred to as direct oral anticoagulants, DOACs), including apixaban, edoxaban, dabigatran and rivaroxaban, are increasingly used for stroke prevention in AF in clinical practice. NOACs have been shown to outperform VKAs, such as warfarin, in reducing risk of recurrent stroke for patients with AF after ischaemic stroke [8–10]. Furthermore, the risk of intracranial bleeding may be substantially lower for most NOACs compared with warfarin, but the risk of gastrointestinal bleeding may be higher [11]. A higher risk of ischaemic stroke has been shown with the use of APA in AF compared to warfarin, but the risk of clinically relevant bleeding may be lower [11]. NOACs are increasingly used in clinical practice to reduce the risk of recurrent stroke or ICH for patients with AF after ischaemic stroke [8–10].

The aims of the current study were to examine the use of OACs and APA after ischaemic stroke for people with AF and carotid artery disease, and determine associations with 1-year mortality, recurrent stroke and major bleeding.

## Methods

A retrospective cohort study was performed by utilising TriNetX, a global federated health research network. TriNetX provided access to statistics on electronic medical records from participating healthcare organisations, predominately in the United States (US). At the time of the analyses, 71 healthcare organisations were included in the TriNetX network and these included academic medical centres, specialty physician practices and community hospitals.

### Inclusion Criteria

The TriNetX online research platform was searched on 15th November 2022 for patients with either (1) first-time stroke due to embolism, thrombosis or unspecified occlusion or stenosis of carotid arteries at least 1 year prior to the search date or (2) first-time ischaemic stroke and carotid artery disease recorded. Patients were only included if they had AF recorded any time before or within 30 days of stroke. The search was limited to patients meeting the inclusion criteria between 1st January 2012 (the year the ESC published guidelines listing NOACs for stroke prevention for AF) and 15th October 2021 (allowing for least 1-year follow-up for all participants). The following International Classification of Diseases, Ninth Revision and Tenth Revision, Clinical Modification (ICD-10-CM) codes were included: I63.03 cerebral infarction due to thrombosis of carotid artery; I63.13 cerebral infarction due to embolism of carotid artery; I63.23 cerebral infarction due to unspecified occlusion or stenosis of carotid arteries or I63 cerebral infarction and I65.2 occlusion and stenosis of carotid artery. The ICD-10-CM code I48 was used to identify AF or flutter. Patients who died within 30 days after ischaemic stroke were not included in analyses. No further exclusion criteria were imposed to limit selection bias.

### Exposures

Four distinct cohorts were examined based on OACs and APA received after ischaemic stroke: (1) NOACs (dabigatran, rivaroxaban, apixaban or edoxaban), (2) NOACs plus APA (dipyridamole, clopidogrel or aspirin), (3) warfarin or (4) warfarin plus APA. The time period examined was 2 to 6 weeks after ischaemic stroke to avoid inclusion of bridging APA which may be routinely offered prior to initiation of OACs. The cohort who received NOACs without use of APA was chosen as the reference group for analyses, except where warfarin and warfarin plus APA use was compared; here, the warfarin cohort without APA use was chosen as the reference group.

### Outcomes

The outcomes were all-cause mortality, recurrent ischaemic stroke (ICD-10-CM code: I63) and major bleeding between 6 weeks and 1 year after the index ischaemic stroke. Major bleeding was defined using the ICD-10-CM codes listed in Supplementary Table 1.

### Statistical Analysis

All statistical analyses were completed on the TriNetX online research platform. The TriNetX platform was used to run 1:1 propensity score matching using logistic regression for each cohort comparison. The platform uses nearest-neighbour matching with tolerance level of 0.01 and difference between propensity scores ≤ 0.1. The following variables were included in propensity score matching: age, sex, ethnicity and the following health conditions identified from ICD-10-CM codes in electronic medical records: hypertensive diseases (I10-I16), ischaemic heart diseases (I20-I25), heart failure (I50), peripheral vascular disease (I73.9), atherosclerosis (I70), diabetes mellitus (E08-E13), liver disease (K70-K77) and acute kidney failure and chronic kidney disease (N17-N19). Furthermore, the presence of cardiac and vascular implants and grafts was also included in propensity score matching including the following: presence of coronary angioplasty implant and grant (Z95.5), presence of cardiac pacemaker (Z95.0) and presence of aortocoronary bypass graft (Z95.1). Standardised mean differences (SMDs) were reported and SMDs <0.1 were considered well balanced. No imputations were made for missing data. Cox proportional hazard models were used to calculate hazard ratios (HRs) with 95% confidence intervals (CIs). Statistical significance was prespecified as *p*<0.05.

## Results

### Patient Characteristics

In total, 5708 patients met the inclusion criteria. Of these patients, 24.1% (*n*=1628) received non-vitamin K antagonist oral anticoagulants (NOACs) with no APA, 26.0% (*n*=1401) received NOACs plus APA, 20.7% (*n*=1243) received warfarin without APA and 29.2% (*n*=1436) received warfarin plus APA. Of the total cohort, 53.8% (*n*=3075) had a diagnosis of AF recorded before the index stroke, and 46.1% (*n*=2633) had no previous diagnosis of AF, but AF was recorded in the 30 days following stroke. Furthermore, 45.0% (*n*=2568) of patients had a recording of receiving OACs within the 6 months prior to the index stroke. The characteristics of the patients by OAC and APA use following stroke are shown in Table 1. After propensity score matching, the cohorts were well balanced for age, sex, ethnicity, co-morbidities and presence of cardiac and vascular implants and grafts (SMDs <0.1 for all comparisons; Supplementary Tables 2, 3, 4, and 5).

**Table 1:** Characteristics of cohorts with carotid disease and atrial fibrillation, by anticoagulant and antiplatelet medication use after ischaemic stroke, before propensity score matching

Characteristic	NOACs without APA (*n*=1628)	NOACs plus APA (*n*=1401)	Warfarin without APA (*n*=1243)	Warfarin plus APA (*n*=1436)
Age, mean (SD)	75.0 (10.4)	74.2 (9.9)	73.4 (11.6)	73.9 (11.7)
Female	46.7 (760)	38.5 (540)	42.9 (533)	47.1 (662)
Ethnicity*				
White	75.0 (1221)	78.9 (1105)	78.2 (972)	79.2 (1138)
Black or African American	13.9 (226)	11.9 (167)	12.5 (155)	11.8 (169)
Unknown	9.8 (159)	7.2 (101)	8.5 (106)	7.9 (113)
Hypertensive diseases	72.9 (1187)	69.5 (973)	94.3 (1172)	96.1 (1380)
Ischemic heart diseases	44.6 (726)	49.4 (692)	68.1 (847)	79.3 (1139)
Heart failure	33.2 (541)	36.0 (504)	59.5 (739)	67.4 (968)
Diabetes mellitus	33.4 (543)	39.3 (550)	50.3 (625)	54.5 (782)
Acute kidney failure and chronic kidney disease	31.6 (514)	35.0 (490)	55.1 (685)	64.9 (932)
Liver disease	11.6 (189)	11.1 (156)	18.6 (231)	19.1 (274)
Atherosclerosis	19.0 (309)	18.8 (263)	31.8 (395)	33.1 (475)
Peripheral vascular disease	14.7 (239)	17.8 (249)	26.6 (331)	27.7 (398)
Presence of aortocoronary bypass graft	9.7 (158)	12.0 (168)	20.4 (254)	27.3 (392)
Presence of coronary angioplasty implant and graft	7.7 (126)	11.3 (159)	10.7 (133)	16.1 (231)
Presence of cardiac pacemaker	7.7 (125)	7.2 (101)	15.6 (194)	13.5 (194)

Values are % (*n*), unless otherwise stated. Medication use based on period 2–6 weeks after ischaemic stroke. *NOAC*, non-vitamin K antagonist oral anticoagulants; *SD*, standard deviation. *Some other ethnicity categories were available, but the numbers were below 10 and cannot be identified

### Anticoagulant and Antiplatelet Agent Use and All-Cause Mortality After Ischaemic Stroke

After propensity score matching, there was no statistically significant difference in 1-year all-cause mortality when comparing use of NOACs without APA to NOACs plus APA or warfarin without APA. However, compared to receiving NOACs without APA, receiving warfarin plus APA was associated with a higher risk of mortality (HR 1.51 (95% CI 1.20, 1.89)). There was no statistically significant difference in 1-year all-cause mortality when comparing use of warfarin with APA and warfarin without APA (Table 2).

**Table 2:** Use of oral anticoagulants and APA after ischaemic stroke and associations with 1-year mortality, recurrent stroke and major bleeding for patients with atrial fibrillation and carotid disease, after propensity score matching

Medication use	Participants, *n*	Deaths, %	HR (95% CI) for mortality	Recurrent stroke, %	HR (95% CI) for stroke	Major bleeding, %	HR (95% CI) for major bleeding
NOACs without APA	1123	11.0	Ref	44.9	Ref	19.4	Ref
Warfarin without APA	1123	13.5	1.19 (0.93, 1.52)	41.9	0.90 (0.79, 1.02)	25.6	1.34 (1.12, 1.60)
NOACs without APA	1112	11.4	Ref	50.0	Ref	20.7	Ref
Warfarin plus APA	1112	17.0	1.51 (1.20, 1.89)	47.9	0.98 (0.87, 1.11)	31.5	1.66 (1.40, 1.96)
NOACs without APA	1269	12.1	Ref	48.7	Ref	18.5	Ref
NOAC plus APA	1269	13.2	1.12 (0.90, 1.40)	46.6	1.01 (0.90, 1.13)	21.9	1.27 (1.07, 1.51)
Warfarin without APA	1089	13.7	Ref	41.5	Ref	28.1	Ref
Warfarin plus APA	1089	15.4	1.17 (0.94, 1.47)	42.2	1.05 (0.92, 1.20)	27.3	0.98 (0.84, 1.15)

*HR*, hazard ratio; *CI*, confidence interval; *NOAC*, non-vitamin K antagonist oral anticoagulants; *Ref*, reference group

### Anticoagulant and Antiplatelet Agent Use and Recurrent Ischaemic Stroke

After propensity score matching, there was no statistically significant difference in risk of recurrent stroke when comparing use of NOACs without APA to NOACs plus APA (HR 1.01 (95% CI 0.90, 1.13)), warfarin with APA (HR 0.98 (95% CI 0.87, 1.11)) or warfarin without APA (HR 0.90 (95% CI (0.79, 1.02)). There was also no statistically significant difference in risk of recurrent stroke when comparing use of warfarin with and without APA (HR 0.98 (95% CI 0.87, 1.11)).

### Anticoagulant and Antiplatelet Agent Use and Major Bleeding After Ischaemic Stroke

Compared to receiving NOACs without APA, receiving warfarin plus APA, warfarin without APA or NOACs plus APA was associated with a higher risk of major bleeding (HRs 1.66 (95% CI 1.40, 1.96), 1.34 (95% CI 1.12, 1.60) and 1.27 (95% CI 1.07, 1.51), respectively). There was no significant difference in the risk of major bleeding when comparing use of warfarin without APA to warfarin plus APA (1.19 (95% CI 0.93, 1.52)).

## Discussion

This study suggests that for people with AF and carotid artery disease, receiving APA in addition to OACs after ischaemic stroke is not associated with a lower risk of 1-year mortality or recurrent stroke, but may be associated with a higher risk of major bleeding. First, compared to the use of NOACs without APA, the use of NOACs plus APA was not significantly associated with the risk of 1-year mortality or recurrent stroke, but was associated with a higher risk of major bleeding. Second, receiving warfarin plus APA was associated with a higher risk of major bleeding and mortality compared to the use of NOACs without APA. Third, receiving warfarin without AF was also associated with a higher risk of major bleeding compared to the use of NOACs without APA. Fourth, there were no statistically significant differences in any of the outcomes examined when comparing the use of warfarin plus APA to the use of warfarin without APA.

There has been relatively little research focused on patients with AF and concomitant carotid atherosclerosis after acute ischaemic stroke. In a long-term study of over 700 patients with AF, carotid atherosclerosis was associated with an increased risk of ischaemic stroke [12]. In a study of 103 ischaemic stroke patients with non-valvular AF, stenosis of the extracranial carotid artery was reported in one in four patients and was associated with worse clinical outcomes for patients [13].

European Society of Cardiology (ESC) guidelines for the management of AF state that there is no evidence that the addition of APA (e.g. clopidogrel, aspirin or dipyridamole) to NOACs improves outcomes for patients with AF after ischaemic stroke [5]. However, there is no specific guidance for those with concomitant carotid disease. In the current study, prolonged use of APA more than 2 weeks following stroke was not significantly associated with a lower risk of recurrent stroke. Furthermore, the use of OACs with APA was associated with a higher risk of major bleeding and the use of warfarin with APA was also associated with a higher risk of mortality compared to the use of NOACs without APA.

A recent retrospective multicentre study of over 2500 patients with acute ischaemic stroke, AF and large artery steno-occlusive disease suggested the use of OACs plus APA was associated with an increased risk of 3-month recurrent stroke, myocardial infarction and all-cause mortality compared with OACs alone [14]. The current study adds to the previous evidence base by further suggesting no beneficial effect of using APA concomitantly with OACs and potential for worse outcomes 1 year following stroke. Previous studies have not compared the use of NOACs versus VKAs in people with AF and carotid artery disease following stroke, and have not examined longer term outcomes. Previous studies have shown that NOACs offer better safety in terms of less intracranial bleeding compared to warfarin in secondary stroke prevention for people with AF [8]. The current study suggests these findings may also extend to people with AF and concomitant carotid artery disease as a significantly higher risk of major bleeding with warfarin compared to NOACs without APA was observed.

There have been no randomised controlled trials (RCTs) comparing OACs plus APA versus OACs without APA for people with AF after ischaemic stroke who have carotid artery disease. Observed associations suggesting no additional benefit of APA with OACs for outcomes at 1 year in people with AF and carotid artery disease following ischaemic stroke would need to be confirmed with evidence from RCTs.

To reduce the risk of recurrent stroke, the majority of patients with AF should be offered OACs. Patients with AF who are not low risk of stroke can be determined with the CHA_2_DS_2_-VASc score: a score of ≥ 1 for men or ≥ 2 for women [15, 16]. As prior stroke history is 2 points on the CHA_2_DS_2_-VASc score, all patients with AF after ischaemic stroke exceed the ‘low risk of stroke’ threshold according to the CHA_2_DS_2_-VASc score, and should be offered OAC unless there is an absolute contraindication. Bleeding risk assessment is also recommended for people with AF before initiation of anticoagulation, with use of a bleeding risk tool such as the HAS-BLED score [17, 18]. A high bleeding risk score alone should not be reason to withhold anticoagulation, but this information can be used to guide the level of patient monitoring required after initiation of anticoagulation and to address the modifiable risk factors [19]. Such an approach to appropriate use of the HAS-BLED score has translated to lower bleeding rates and an increase in OAC use, when investigated in a prospective trial [20], contributing to overall better outcomes [21].

Evidence from large cohort studies has suggested early introduction of OACs at a median of 4 days after ischaemic stroke, and to a lesser extent APA, is associated with lower recurrent stroke without increased risk of ICH, compared to later initiation [22, 23]. Furthermore, early initiation of NOACs after ischaemic stroke compared to VKA has been associated with a lower risk of poor clinical outcomes with no increased risk of adverse events [24–28]. Research into the optimal timing for initiation of OACs for people with AF following ischaemic stroke is ongoing [29]. Early use of OACs within 48 h after ischaemic stroke for patients with AF may increase the risk of symptomatic ICH without significantly reducing the risk of recurrent ischaemic stroke [30]. Initiation of NOACs within 4–14 days of stroke in patients with AF is associated with a lower risk of 90-day recurrent stroke and outweighs the risk of symptomatic ICH [23, 28, 31]. The current study could not further examine the optimal timing of OAC initiation for people with AF and carotid artery disease following stroke.

### Strengths and Limitations

The current study utilises a global research network and includes a large cohort of patients with AF, stroke and carotid artery disease. The network includes data on patient demographics and co-morbidities which were included in propensity score matching.

The study has several limitations of note. Only medication use during the first 6 weeks following stroke was examined in this study, and therefore, the impact of changes to OACs or APA after the first 6 weeks following stroke was not taken into account in the analyses. Furthermore, the duration of APA use and overlap with OACs is a limitation, although we have attempted to exclude the use of bridging APA by not including use in the first 2 weeks after ischaemic stroke. Dosage of the OACs may have an important impact on outcomes, but this could not be explored within the current dataset. With the data available, the degree of stenosis or whether the carotid disease was ipsilateral to the index infarct could not be determined. Furthermore, data on vascular imaging were not available. Although the cohorts were propensity score–matched for age, sex, ethnicity, many co-morbidities and presence of cardiac and vascular implants and grafts, residual confounding factors may include variables which were not available in the research network, such as stroke severity, lifestyle factors (e.g. alcohol consumption, levels of physical activity, smoking) and genetic factors. In the current study, outcomes including mortality, recurrent stroke and major bleeding were examined, but some outcomes of interest following the index stroke such as functional status were not available. The data were collected from electronic medical records of participating healthcare organisations, and outcomes which occur outside of the organisations are not well captured.

Defining stroke and other health conditions was based on ICD codes and no further information was available about how the conditions were diagnosed. The rates of recurrent stroke in this study were high compared to those in previous studies [32]. However, rates of recurrent stroke at 1 year specifically in a population with AF and carotid artery disease are unclear. In a recent post hoc analysis of a RCT, rates of 90-day recurrent stroke for patients with minor stroke and symptomatic carotid stenosis were high at 23.5%, and the current cohort is high risk with AF and carotid artery disease and follow-up was up to 1 year [33].

Previous research has suggested the recording of health conditions using ICD codes may vary by patient factors including age, co-morbidities, severity of illness, length of stay in hospital and in-hospital mortality [34]. The use of other medicines following stroke may also impact patient outcomes, but within the TriNetX platform it was not possible to account for the use of other medicines received. The impact of attending different healthcare organisations could also not be determined due to data privacy restrictions. The TriNetX network includes multiple healthcare organisations in the US but the results may not be representative of the wider US population and the generalisability of the results beyond this population is unclear.

## Conclusions

The current study provides observational evidence to suggest that the use of APA in addition to OACs is not associated with improved outcomes at 1 year for patients with AF and carotid artery disease after ischaemic stroke. The use of OACs with APA may also be associated with a higher risk of worse outcomes at 1 year such as major bleeding. There is no evidence from RCTs to support the use of APA in addition to OACs for recurrent stroke prevention in patients with AF and carotid artery disease. RCT evidence would be needed to confirm optimal recurrent stroke prevention strategies for people with AF and carotid artery disease following stroke.

### Supplementary Information

ESM 1 (DOCX 20 kb)

## Data Availability

To gain access to the data in the TriNetX research network, a request can be made to TriNetX (https://live.trinetx.com), but costs may be incurred, a data sharing agreement would be necessary and no patient identifiable information can be obtained.

## Code Availability

Not applicable. All analyses were completed on the TriNetX online research platform.

## References

[1] Lip G, Freedman B, De Caterina R, Potpara TS. Stroke prevention in atrial fibrillation: past, present and future. Comparing the guidelines and practical decision-making. Thromb Haemost. 2017;117(7):1230–9. 28597905 10.1160/TH16-11-0876

[2] Lip GYH, Banerjee A, Boriani G, et al. Antithrombotic therapy for atrial fibrillation: CHEST Guideline and Expert Panel Report. Chest. 2018;154(5):1121–201. 30144419 10.1016/j.chest.2018.07.040

[3] Lehtola H, Airaksinen KEJ, Hartikainen P, et al. Stroke recurrence in patients with atrial fibrillation: concomitant carotid artery stenosis doubles the risk. Eur J Neurol. 2017;24(5):719–25. 28317289 10.1111/ene.13280

[4] Abbott AL. Medical (nonsurgical) intervention alone is now best for prevention of stroke associated with asymptomatic severe carotid stenosis: results of a systematic review and analysis. Stroke. 2009;40(10):e573–83. 19696421 10.1161/STROKEAHA.109.556068

[5] Hindricks G, Potpara T, Dagres N, et al. ESC Guidelines for the diagnosis and management of atrial fibrillation developed in collaboration with the European Association of Cardio-Thoracic Surgery (EACTS). Eur Heart J. 2020;2020.

[6] Lee SR, Rhee TM, Kang DY, et al. Meta-analysis of oral anticoagulant monotherapy as an antithrombotic strategy in patients with stable coronary artery disease and nonvalvular atrial fibrillation. Am J Cardiol. 2019;124(6):879–85. 31311662 10.1016/j.amjcard.2019.05.072

[7] Malhotra K, Gornbein J, Saver JL. Ischemic strokes due to large-vessel occlusions contribute disproportionately to stroke-related dependence and death: a review. Front Neurol. 2017;8:651. 29250029 10.3389/fneur.2017.00651 PMC5715197

[8] Ntaios G, Papavasileiou V, Diener HC, Makaritsis K, Michel P. Nonvitamin-K-antagonist oral anticoagulants versus warfarin in patients with atrial fibrillation and previous stroke or transient ischemic attack: an updated systematic review and meta-analysis of randomized controlled trials. Int J Stroke. 2017;12(6):589–96. 28730948 10.1177/1747493017700663

[9] Hohmann C, Hohnloser SH, Jacob J, et al. Non-vitamin K oral anticoagulants in comparison to phenprocoumon in geriatric and non-geriatric patients with non-valvular atrial fibrillation. Thromb Haemost. 2019;119(6):971–80. 30900223 10.1055/s-0039-1683422

[10] Hohnloser SH, Basic E, Nabauer M. Changes in oral anticoagulation therapy over one year in 51,000 atrial fibrillation patients at risk for stroke: a practice-derived study. Thromb Haemost. 2019;119(6):882–93. 30900220 10.1055/s-0039-1683428

[11] López-López JA, Sterne JAC, Thom HHZ, et al. Oral anticoagulants for prevention of stroke in atrial fibrillation: systematic review, network meta-analysis, and cost effectiveness analysis. BMJ. 2017;359:j5058. 29183961 10.1136/bmj.j5058 PMC5704695

[12] Bekwelem W, Jensen PN, Norby FL, et al. Carotid atherosclerosis and stroke in atrial fibrillation: the Atherosclerosis Risk in Communities study. Stroke. 2016;47(6):1643–6. 27217511 10.1161/STROKEAHA.116.013133 PMC4880451

[13] Chang YJ, Ryu SJ, Lin SK. Carotid artery stenosis in ischemic stroke patients with nonvalvular atrial fibrillation. Cerebrovasc Dis. 2002;13(1):16–20. 11810005 10.1159/000047740

[14] Kim JT, Lee JS, Kim BJ, et al. Effectiveness of adding antiplatelets to oral anticoagulants in patients with acute ischemic stroke with atrial fibrillation and concomitant large artery steno-occlusion. Transl Stroke Res. 2020;11(6):1322–31. 32472251 10.1007/s12975-020-00822-z

[15] Lip GY, Nieuwlaat R, Pisters R, Lane DA, Crijns HJ. Refining clinical risk stratification for predicting stroke and thromboembolism in atrial fibrillation using a novel risk factor-based approach: the euro heart survey on atrial fibrillation. Chest. 2010;137(2):263–72. 19762550 10.1378/chest.09-1584

[16] Lip Gregory YH, Nielsen PB. Should patients with atrial fibrillation and 1 stroke risk factor (CHA2DS2-VASc score 1 in men, 2 in women) be anticoagulated? Circulation. 2016;133(15):1498–503. 27067084 10.1161/CIRCULATIONAHA.115.016713

[17] Lip GY, Frison L, Halperin JL, Lane DA. Comparative validation of a novel risk score for predicting bleeding risk in anticoagulated patients with atrial fibrillation: the HAS-BLED (Hypertension, Abnormal Renal/Liver Function, Stroke, Bleeding History or Predisposition, Labile INR, Elderly, Drugs/Alcohol Concomitantly) score. J Am Coll Cardiol. 2011;57(2):173–80. 21111555 10.1016/j.jacc.2010.09.024

[18] Lip GY, Andreotti F, Fauchier L, et al. Bleeding risk assessment and management in atrial fibrillation patients: a position document from the European Heart Rhythm Association, endorsed by the European Society of Cardiology Working Group on Thrombosis. Europace. 2011;13(5):723–46. 21515596 10.1093/europace/eur126

[19] Lip GYH, Lane DA. Assessing bleeding risk in atrial fibrillation with the HAS-BLED and ORBIT scores: clinical application requires focus on the reversible bleeding risk factors. Eur Heart J. 2015;36(46):3265–7. 26351397 10.1093/eurheartj/ehv415

[20] Guo Y, Lane DA, Chen Y, Lip GYH. Regular bleeding risk assessment associated with reduction in bleeding outcomes: the mAFA-II randomized trial. Am J Med. 2020;133(10):1195–202.e2. 32289310 10.1016/j.amjmed.2020.03.019

[21] Guo Y, Guo J, Shi X, et al. Mobile health technology-supported atrial fibrillation screening and integrated care: a report from the mAFA-II trial Long-term Extension Cohort. Eur J Intern Med. 2020. 10.1016/j.ejim.2020.09.024 PMC7553102 33067121

[22] Abdul-Rahim AH, Fulton RL, Frank B, et al. Association of improved outcome in acute ischaemic stroke patients with atrial fibrillation who receive early antithrombotic therapy: analysis from VISTA. Eur J Neurol. 2015;22(7):1048–55. 25319957 10.1111/ene.12577

[23] Paciaroni M, Agnelli G, Falocci N, et al. Early recurrence and major bleeding in patients with acute ischemic stroke and atrial fibrillation treated with non-vitamin-K oral anticoagulants (RAF-NOACs) study. J Am Heart Assoc. 2017;6(12). 10.1161/JAHA.117.007034 PMC5779022 29220330

[24] Seiffge DJ, Paciaroni M, Wilson D, et al. Direct oral anticoagulants versus vitamin K antagonists after recent ischemic stroke in patients with atrial fibrillation. Ann Neurol. 2019;85(6):823–34. 30980560 10.1002/ana.25489 PMC6563449

[25] Macha K, Volbers B, Bobinger T, et al. Early initiation of anticoagulation with direct oral anticoagulants in patients after transient ischemic attack or ischemic stroke. J Stroke Cerebrovasc Dis. 2016;25(9):2317–21. 27449113 10.1016/j.jstrokecerebrovasdis.2016.06.031

[26] Cappellari M, Carletti M, Danese A, Bovi P. Early introduction of direct oral anticoagulants in cardioembolic stroke patients with non-valvular atrial fibrillation. J Thromb Thrombolysis. 2016;42(3):393–8. 27329483 10.1007/s11239-016-1393-9

[27] Coleman CI, Peacock WF, Bunz TJ, Alberts MJ. Effectiveness and safety of apixaban, dabigatran, and rivaroxaban versus warfarin in patients with nonvalvular atrial fibrillation and previous stroke or transient ischemic attack. Stroke. 2017;48(8):2142–9. 28655814 10.1161/STROKEAHA.117.017474

[28] Arihiro S, Todo K, Koga M, et al. Three-month risk-benefit profile of anticoagulation after stroke with atrial fibrillation: the SAMURAI-Nonvalvular Atrial Fibrillation (NVAF) study. Int J Stroke. 2016;11(5):565–74. 26927811 10.1177/1747493016632239

[29] OPTIMAS: OPtimal TIMing of Anticoagulation After Acute Ischaemic Stroke: a randomised controlled trial. https://ClinicalTrials.gov/show/NCT03759938. https://ClinicalTrials.gov/show/NCT03759938.

[30] Paciaroni M, Agnelli G, Micheli S, Caso V. Efficacy and safety of anticoagulant treatment in acute cardioembolic stroke. Stroke. 2007;38(2):423–30. 17204681 10.1161/01.STR.0000254600.92975.1f

[31] Seiffge DJ, Traenka C, Polymeris A, et al. Early start of DOAC after ischemic stroke. Risk of intracranial hemorrhage and recurrent events. Neurology. 2016;87(18):1856–62. 27694266 10.1212/WNL.0000000000003283

[32] Diener H-C, Hankey GJ, Easton JD, et al. Non-vitamin K oral anticoagulants for secondary stroke prevention in patients with atrial fibrillation. Eur Heart J Suppl. 2020;22(Supplement_I):I13–21. 33093818 10.1093/eurheartj/suaa104 PMC7556747

[33] Yaghi S, de Havenon A, Rostanski S, et al. Carotid stenosis and recurrent ischemic stroke: a post-hoc analysis of the POINT trial. Stroke. 2021;52(7):2414–7. 33940954 10.1161/STROKEAHA.121.034089 PMC8268075

[34] Chong WF, Ding YY, Heng BH. A comparison of comorbidities obtained from hospital administrative data and medical charts in older patients with pneumonia. BMC Health Serv Res. 2011;11:105. 21586172 10.1186/1472-6963-11-105 PMC3112394

