# Supplementary material for: Antiplatelet Agents and Oral Anticoagulant Use in Patients with Atrial Fibrillation and Carotid Artery Disease After First-Time Ischaemic Stroke
Source: Cardiovasc Drugs Ther. 2023 Jan 24;38(4):731–7. doi: 10.1007/s10557-023-07433-4 (PMC11266273; doi:10.1007/s10557-023-07433-4)
Supplement: Supplementary file 1 — (DOCX 20 kb) [file 10557_2023_7433_MOESM1_ESM.docx]

**Supplementary Table 1. List of ICD-10-CM codes used to define major bleeding**

| D62: acute post-haemorrhagic anaemia  H11.3: conjunctival haemorrhage  H35.6: retinal haemorrhage  H43.1: vitreous haemorrhage  I60: subarachnoid haemorrhage  I61: intracerebral haemorrhage  I62: other non-traumatic intracranial haemorrhage  J94.2: haemothorax  K25.0: gastric ulcer acute with haemorrhage  K25.2: gastric ulcer acute with haemorrhage and perforation  K25.4: gastric ulcer chronic or unspecified with haemorrhage  K26.0: duodenal ulcer acute with haemorrhage  K26.2: duodenal ulcer acute with haemorrhage and perforation  K26.4: duodenal ulcer chronic or unspecified with haemorrhage  K27.0: peptic ulcer acute with haemorrhage  K27.2: peptic ulcer acute with haemorrhage and perforation  K27.4: peptic ulcer chronic or unspecified with haemorrhage  K28.0: gastrojejunal ulcer: acute with haemorrhage  K28.2: gastrojejunal ulcer acute with haemorrhage and perforation  K29.0: acute haemorrhagic gastritis  N02: recurrent and persistent haematuria  R04: haemorrhage from respiratory passages  R31: unspecified haematuria  R58: haemorrhage, not classified elsewhere  S06.3: focal brain injury  S06.4: epidural haemorrhage  S06.5: traumatic subdural haemorrhage  S06.6: traumatic subarachnoid haemorrhage |
| --- |

**Supplementary Table 2. Characteristics of cohorts with carotid disease and atrial fibrillation who received NOACs without APA or warfarin without APA after ischaemic stroke, after propensity score matching.**

| **Characteristic** | **NOACs without APA**  **(n=1,123)** | **Warfarin without APA**  **(n=1,123)** | **SMD** |
| --- | --- | --- | --- |
| Age, mean (SD) | 74.0 (10.6) | 73.9 (11.4) | 0.012 |
| Female | 44.5 (500) | 45.0 (505) | 0.009 |
| Ethnicity* |  |  |  |
| White | 77.6 (871) | 77.6 (871) | <0.001 |
| Black or African American | 13.1 (147) | 12.7 (143) | 0.011 |
| Unknown | 8.6 (97) | 8.8 (99) | 0.006 |
| Hypertensive diseases | 93.9 (1,054) | 93.8 (1,053) | 0.004 |
| Ischemic heart diseases | 64.8 (728) | 65.3 (733) | 0.009 |
| Heart failure | 56.2 (631) | 55.7 (625) | 0.011 |
| Diabetes mellitus | 48.2 (541) | 47.8 (537) | 0.007 |
| Acute kidney failure and chronic kidney disease | 52.2 (586) | 51.6 (579) | 0.012 |
| Liver disease | 16.7 (188) | 17.1 (192) | 0.010 |
| Atherosclerosis | 30.1 (338) | 30.8 (346) | 0.015 |
| Peripheral vascular disease | 22.6 (254) | 22.7 (255) | 0.002 |
| Presence of aortocoronary bypass graft | 16.7 (187) | 16.8 (189) | 0.005 |
| Presence of coronary angioplasty implant and graft | 10.3 (116) | 11.0 (124) | 0.023 |
| Presence of cardiac pacemaker | 13.7 (154) | 13.6 (153) | 0.003 |

Values are % (n), unless otherwise stated. NOAC: non-vitamin K antagonist oral anticoagulants; SD: standard deviation; SMD: standardised mean difference. *Some other ethnicity categories were available, but the numbers were below 10 and cannot be identified.

**Supplementary Table 3. Characteristics of cohorts with carotid disease and atrial fibrillation who received NOACs without APA or warfarin plus APA after ischaemic stroke, after propensity score matching.**

| **Characteristic** | **NOACs without APA**  **(n=1,112)** | **Warfarin plus APA**  **(n=1,112)** | **SMD** |
| --- | --- | --- | --- |
| Age, mean (SD) | 73.2 (10.7) | 73.6 (10.2) | 0.037 |
| Female | 39.7 (442) | 40.5 (450) | 0.015 |
| Ethnicity* |  |  |  |
| White | 77.2 (859) | 77.9 (866) | 0.015 |
| Black or African American | 13.0 (145) | 12.3 (137) | 0.022 |
| Unknown | 8.7 (97) | 8.6 (96) | 0.003 |
| Hypertensive diseases | 74.2 (825) | 74.6 (829) | 0.008 |
| Ischemic heart diseases | 54.6 (607) | 55.3 (615) | 0.014 |
| Heart failure | 41.7 (464) | 42.5 (473) | 0.016 |
| Diabetes mellitus | 36.3 (404) | 37.9 (422) | 0.034 |
| Acute kidney failure and chronic kidney disease | 38.7 (430) | 39.3 (437) | 0.013 |
| Liver disease | 11.8 (131) | 12.0 (133) | 0.006 |
| Atherosclerosis | 20.4 (227) | 21.0 (233) | 0.013 |
| Peripheral vascular disease | 17.4 (193) | 18.1 (201) | 0.019 |
| Presence of aortocoronary bypass graft | 12.7 (141) | 13.7 (152) | 0.029 |
| Presence of coronary angioplasty implant and graft | 8.9 (99) | 9.4 (104) | 0.016 |
| Presence of cardiac pacemaker | 8.6 (96) | 8.9 (99) | 0.010 |

Values are % (n), unless otherwise stated. NOAC: non-vitamin K antagonist oral anticoagulants; SD: standard deviation; SMD: standardised mean difference. *Some other ethnicity categories were available, but the numbers were below 10 and cannot be identified.

**Supplementary Table 4. Characteristics of cohorts with carotid disease and atrial fibrillation who received NOACs without APA or NOAC plus APA after ischaemic stroke, after propensity score matching.**

| **Characteristic** | **NOACs without APA**  **(n=1,269)** | **NOACs plus APA**  **(n=1,269)** | **SMD** |
| --- | --- | --- | --- |
| Age, mean (SD) | 74.4 (10.4) | 74.4 (9.9) | 0.001 |
| Female | 40.0 (507) | 41.4 (526) | 0.030 |
| Ethnicity* |  |  |  |
| White | 40.0 (507) | 41.4 (526) | 0.036 |
| Black or African American | 13.5 (171) | 12.4 (157) | 0.033 |
| Unknown | 8.2 (104) | 7.8 (99) | 0.015 |
| Hypertensive diseases | 69.7 (885) | 69.1 (877) | 0.014 |
| Ischemic heart diseases | 46.6 (591) | 46.5 (590) | 0.002 |
| Heart failure | 34.0 (431) | 34.0 (432) | 0.002 |
| Diabetes mellitus | 36.6 (465) | 35.6 (452) | 0.021 |
| Acute kidney failure and chronic kidney disease | 32.9 (417) | 32.8 (416) | 0.002 |
| Liver disease | 10.8 (137) | 11.3 (143) | 0.015 |
| Atherosclerosis | 18.8 (238) | 18.7 (237) | 0.002 |
| Peripheral vascular disease | 16.4 (208) | 16.3 (207) | 0.002 |
| Presence of aortocoronary bypass graft | 10.6 (134) | 10.9 (138) | 0.010 |
| Presence of coronary angioplasty implant and graft | 8.7 (111) | 9.3 (118) | 0.019 |
| Presence of cardiac pacemaker | 7.1 (90) | 7.0 (89) | 0.003 |

Values are % (n), unless otherwise stated. NOAC: non-vitamin K antagonist oral anticoagulants; SD: standard deviation; SMD: standardised mean difference. *Some other ethnicity categories were available, but the numbers were below 10 and cannot be identified.

**Supplementary Table 5. Characteristics of cohorts with carotid disease and atrial fibrillation who received warfarin without APA or warfarin plus APA after ischaemic stroke, after propensity score matching.**

| **Characteristic** | **Warfarin without APA**  **(n=1,089)** | **Warfarin plus antiplatelet**  **(n=1,089)** | **SMD** |
| --- | --- | --- | --- |
| Age, mean (SD) | 73.0 (11.8) | 72.8 (10.8) | 0.020 |
| Female | 40.3 (439) | 40.4 (440) | 0.002 |
| Ethnicity* |  |  |  |
| White | 78.3 (853) | 78.4 (854) | 0.002 |
| Black or African American | 12.5 (136) | 12.6 (137) | 0.003 |
| Unknown | 8.4 (91) | 8.1 (88) | 0.010 |
| Hypertensive diseases | 94.8 (1,032) | 94.9 (1,034) | 0.008 |
| Ischemic heart diseases | 72.9 (794) | 73.6 (801) | 0.015 |
| Heart failure | 62.6 (682) | 63.0 (686) | 0.008 |
| Diabetes mellitus | 52.2 (569) | 51.6 (562) | 0.013 |
| Acute kidney failure and chronic kidney disease | 59.7 (650) | 58.5 (637) | 0.024 |
| Liver disease | 22.5 (245) | 22.6 (246) | 0.002 |
| Atherosclerosis | 12.1 (132) | 11.7 (127) | 0.014 |
| Peripheral vascular disease | 14.8 (161) | 15.1 (164) | 0.008 |
| Presence of aortocoronary bypass graft | 18.7 (204) | 18.9 (206) | 0.005 |
| Presence of coronary angioplasty implant and graft | 32.6 (355) | 33.0 (359) | 0.008 |
| Presence of cardiac pacemaker | 27.4 (298) | 28.1 (306) | 0.016 |

Values are % (n), unless otherwise stated. SD: standard deviation; SMD: standardised mean difference. *Some other ethnicity categories were available, but the numbers were below 10 and cannot be identified.
